# Evidence for SIRT1 Mediated HMGB1 Release From Kidney Cells in the Early Stages of Hemorrhagic Shock

**DOI:** 10.3389/fphys.2019.00854

**Published:** 2019-07-05

**Authors:** Siqi Xu, Zhenhua Zeng, Ming Zhao, Qiaobing Huang, Youguang Gao, Xingui Dai, Jiayin Lu, Weiqing Huang, Keseng Zhao

**Affiliations:** ^1^Guangdong Provincial Key Laboratory of Shock and Microcirculation, School of Basic Medical Sciences, Southern Medical University, Guangzhou, China; ^2^Department of Pathology, Qingdao Municipal Hospital (Group), Qingdao, China; ^3^Department of Critical Care Medicine, Nanfang Hospital, Southern Medical University, Guangzhou, China; ^4^Department of Anesthesiology, The First Affiliated Hospital of Fujian Medical University, Fuzhou, China; ^5^Department of Critical Care Medicine, The First People’s Hospital of Chenzhou, Institute of Translational Medicine, Chenzhou, China

**Keywords:** HMGB1, acetylation, sirtuin 1, renal injury, polydatin, hemorrhagic shock

## Abstract

**Background:**

This study is to explore the effect of SIRT1 deacetylating inactivation on organ-derived high mobility group box 1 (HMGB1) during the development of severe hemorrhagic shock (HS).

**Methods:**

Hemorrhagic shock model was reproduced in rats by blood shedding and mimicked in HK-2 cells by hypoxia-reoxygenation (H/R) treatment. The level and acetylation of HMGB1 and the expression and activity of SIRT1 were detected in tissue, serum and cultured cells and supernatant. The deacetylated sites of HMGB1 was determined by Co-IP.

**Results:**

Serum HMGB1 in HS rats was increased but were reduced in multiple organs, especially in kidney tissue, with enhanced HMGB1 acetylation, and reduced deacetylase SIRT1 activity in isolated RTECs. HMGB1 in suspension of H/R-treated HK-2 cells was increased, accompanying with enhanced HMGB1 acetylation, and nuclear-plasma translocation. SIRT1 down-regulated by siRNA aggravated acetylation of HMGB1 and nucleus-to-cytoplasm translocation and resulted in increased HMGB1 in cultured HK-2 suspension. Immunoprecipitation data suggested that SIRT1-indcuced deacetylated sites of HMGB1 were K90 and K177 following H/R. SIRT1 overexpression inhibited the acetylation of HMGB1 and reduced the content of extracellular HMGB1 in H/R-treated HK-2 cells. Inhibiting mutation of SIRT1 restored the acetylation of HMGB1 and HMGB1 content in extracellular suspension. In HS rat model, the neutralization of HMGB1 with antibody could reduce serum HMGB1 and pro-inflammatory cytokine contents, but had no effect on SIRT1 protein expression and activity. Polydatin (PD), a potential SIRT1 agonist, up-regulated SIRT1 activity and inhibited nucleus-to-cytoplasm translocation of HMGB1 in RTECs. Moreover, PD also attenuated RTEC apoptosis, protected renal function, and prolonged survival in HS rats. These beneficial effect of PD is largely blocked by specific inhibition of SIRT1 with Ex527.

**Conclusion:**

The acetylation of HMGB1 in K99 and K177 is enhanced during HS due to the downregulation of SIRT1. The nucleus-to-cytoplasm translocation and the release of acetylated HMGB1 from RTECs of kidney might exacerbate the pro-inflammatory effects of HMGB1 during the development of HS.

## KEY POINTS

- Question: What is the effect of inactivation of deacetylase SIRT1 on organ-derived HMGB1 during the development of severe hemorrhagic shock?
- Findings: The nuclear-cytoplasmic translocation and extracellular secretion of acetylated HMGB1 in renal tubular epithelial cells following hemorrhagic shock was mediated by SIRT1 deacetylating inactivation.
- Meaning: Targeting at the deacetylation of organ-derived HMGB1 may be a novel way for hemorrhagic shock treatment.

## Background

Hemorrhagic shock is a pathologic process caused by insufficient perfusion in multiple organs, and usually initiates a systemic post-traumatic inflammatory response. The resulting increased inflammatory response may accelerate the multiple organ dysfunctions (Qiang et al., 2013). HMGB1 protein (HMGB1), also known as high-mobility group protein 1 or amphoterin, is a protein encoded by HMGB1 gene in humans. Extracellular HMGB1 was found to mediate inflammation during sterile and infectious injury and to contribute significantly to disease pathogenesis (Hwang et al., 2014, 2015; Rickenbacher et al., 2014; Rabadi et al., 2015). However, the exact role of HMGB1-mediated inflammation in HS is not fully understood.

It was reported that acetylation promoted the activation of HMGB1 and HMGB1-mediated inflammation in ischemia/reperfusion animal models and other diseases (Hwang et al., 2014, 2015; Rickenbacher et al., 2014; Rabadi et al., 2015). Recently, we confirmed that silent information regulator 2 related enzyme 1 (also known as SIRT1, a typical type III histone deacetylase, can deacetylate multiple histones and non-histone substances [e.g., mitochondrial manganese-dependent superoxide dismutase 2 (SOD2) (Zeng et al., 2015)], and the apoptosis-related protein, p53). Thus, we hypothesize that the acetylation of HMGB1 due to the inhibition of SIRT1 exert a role in the pathogenesis of HS.

We also confirmed that Polydatin (PD; 3,4′,5-trihydroxystibene-3- monoglucoside), isolated from traditional Chinese medical herb Polygonum Cuspidatum, had a similar effect as its analog, resveratrol and worked as a potential SIRT1 agonist (Wang et al., 2013; Li et al., 2015; Zeng et al., 2015, 2016a; Xu et al., 2016). We speculate that PD could activate SIRT1 and inhibit ac-HMGB1 release following HS. In this study, we have tested our hypothesis in both animal model of HS as well as cellular model of hypoxia/re-oxygenation (H/R) using human proximal tubular epithelial-2 (HK-2) cells.

## Materials and Methods

### Reagents and Antibodies

The ELISA kit was obtained from Elabscience Biotechnology Co., Ltd. (Wuhan, Hubei, China). Antibodies against HMGB1 and SIRT1, and siRNA against SIRT1 were obtained from Santa Cruz Biotechnology (Santa Cruz, CA, United States). Antibodies against ac-lysine and PVDF membranes were obtained from Millipore (Billerica, MA, United States). Antibodies against ac-lysine and GAPDH were purchased from CST (Danvers, MA, United States). Antibodies against exact ac-lysine sites (K28, K29, K30, K90, and K177) of HMGB1 were obtained by ABclonal Biotechnology (Wuhan, Hubei, China). For the K28 site, 24-40aa synthetic modified peptide REEH (acetyl-K) KKHPDASVNFSE-C and control peptide REEHKKKHPDASVNFSE-C were selected for antibody preparation; For the K29 site, 24-40aa synthetic modified peptide REEHK (acetyl-K) KHPDASVNFSE-C and control peptide REEHKKKHPDASVNFSE-C were selected for antibody preparation; For the K30 site, the 24-40aa synthetic modified peptide REEHKK (acetyl-K) HPDASVNFSE-C, and the control peptide REEHKKKHPDASVNFSE-C were selected for antibody preparation. For the K90 site, 84–100aa synthetic modified peptide ETKKKF (acetyl-K) DPNAPKRPPS-C, and control peptide ETKKKFKDPNAPKRPPS-C were selected for antibody preparation. For the K177 site, the 167–183aa synthetic modified peptide C-KPDAAKKGVV (acetyl-K) AEKSKK and the control peptide C-KPDAAKKGVVKAEKSKK were selected for antibody preparation. Nuclear and Cytoplasmic Extraction Reagents were obtained from ThermoFisher (Miami, FL, United States). IP kits were purchased from Proteintech Co. (Chicago, IL, United States). SIRT1 Deacetylase Fluorometric Assay kits were obtained from Cyclex (Nagano, Japan). A catalytic mutant of SIRT1 (SIRT1H363Y) lacking deacetylase activity was obtained from Addgene Corporation (Cambridge, MA, United States). FITC annexin V apoptosis detection kits were purchased from BD Biosciences (San Jose, CA, United States). ELISA kits for inflammatory cytokines (TNF-α, IL-1β, and IL-6) were obtained from Dakewe Biotech Company (Shenzhen, Guangdong, China). PD of >99.5% purity was supplied by Neptunus Co. (Shenzhen, Guangdong, China). HMGB1 neutralizing antibody and other chemicals were obtained from Sigma (St. Louis, MO, United States).

### Measurement of Serum HMGB1

Serial blood samples were collected at indicated time points for HS rats (0, 2, 4, 8, and 24 following HS). Levels of HMGB1 were detected by ELISA kit according to the manufacturer’s instructions.

### Establishment of HS Model in Rats

The present study was carried out in strict accordance with the recommendations in the Guide for the Care and Use of Laboratory Animals (US National Institutes of Health, Bethesda, MD, United States). The study protocol was approved by the Committee on Ethics in Animal Experiments of Southern Medical University. In total, 32 specific pathogen-free male Sprague-Dawley rats weighing 180–220 g were used in this study. The rats were housed in plastic cages with a controlled temperature of 25°C, humidity of 50–55%, and a 12 h light/dark cycle. All the animals had free access to food and regular water. All rats were anesthetized and maintained with 1–1.5 MAC isoflurane (flow control at 0.4 L/min) by an animal anesthesia machine (RWD Lifescience, Shenzhen, China) according to the conventional method of the laboratory with slight modifications (Zeng et al., 2015, 2016a). Rats were then subjected to HS for 120 min followed by resuscitation with shed blood. Briefly, after implantation of PE-50 catheters in arterial and venous passages, the MAP was recorded (PowerLAB; AD Instruments, Sydney, NSW, Australia). Rats were bled through a syringe to obtain a MAP of 30 mmHg within 10 min. MAP was maintained for the next 120 min by withdrawal or reinfusion of stored blood. Next, chemical agents including SIRT1 agonist PD, SIRT1 selective inhibitor Ex527, and HMGB1 neutralizing antibody were administered i.v. alone or in combination within 10 min. Shed blood was then reinfused. Animals were then randomly divided into the following four groups: (1) Sham group: rats were anesthetized and underwent surgery without HS attack and drug administration; (2) HS + vehicle group: rats were subjected to HS to maintain a MAP at 30 mmHg for 120 min, followed by the administration of a vehicle (0.3 mL vehicle) and an infusion of shed blood; (3) HS + PD group: rats were subjected to HS to maintain a MAP at 30 mmHg for 120 min, followed by PD administration (30 mg/kg) dissolved in 0.3 mL vehicle, and an infusion of shed blood (Zeng et al., 2016a); (4) HS + PD + Ex527 group: rats were subjected to HS to maintain a MAP at 30 mmHg for 120 min, followed by the administration of PD and Ex527 (5 mg/kg) in 0.3 mL vehicle, and an infusion of shed blood.

### Isolation of RTECs

Rats (eight per group) were killed by cervical dislocation 2 h after the shed blood was reinfused, then kidney tissue was extracted. Briefly, the cortex was cut into fragments, and cells were dissociated by incubation with 1 mg/mL type-I collagenase for 30 min at 37°C. Red blood cells were removed by lysis. RTECs were separated by Percoll gradient density centrifugation. RTEC purity was determined by immunostaining with cytokeratin-18 and Hoechst dye (Zeng et al., 2016a).

### Measurement of HMGB1, ac-HMGB1, SIRT1 With Western Blotting

Both tissues (kidney, lung, and gut) and isolated RTECs and peritoneal macrophages were centrifuged at 14,000rpm for 10 min after homogenization in RIPA lysis buffer, and the clear supernatants were collected. Total protein concentrations in the supernatants were determined by the BCA method. Then, the protein was boiled at 98°C for 5–10 min and stored at -80°C for later analysis. Equal amounts of protein samples were electrophoresed through a 7.5% SDS-polyacrylamide gel and then transferred onto PVDF membrane using wet transfer at 100 V for 90 min at 4°C. Non-specific binding sites were blocked by 5% BSA in 0.05% Tween-20 TBST and then incubated overnight at 4°C with primary antibodies. After incubation with primary antibodies (against HMGB1, SIRT1, and GAPDH) and secondary antibodies, protein bands were detected using chemiluminescence detection reagents. GAPDH was used as an internal reference. ac-HMGB1 levels on IP HMGB1 protein were measured. In this experiment, ACE-lysine antibody was used to deposit the protein complex bound to ACE-lysine on the beads by AminoLink Plus resin beads; the protein samples obtained by mild elution were used to detect Ac-HMGB1 levels by ELISA. The absorbance was measured at 450 nm with a microplate reader within 30 min after dilution according to the instructions. The protein concentration of the corresponding Ac-HMGB1 was converted according to the standard curve. Band intensity was quantified by scanning densitometry. Each measurement was made at least 3 times for each group.

### Immunohistochemistry of Kidney Tissues Isolated From Rats

The kidney tissue was fixed in neutral-buffered formalin, embedded in paraffin, and cut into 4 mm thick transverse sections for immunohistochemistry studies. The expression of SIRT1 in tissues was visualized using the immunohistochemical Envison method (Dako, Copenhagen, Denmark) with goat polyclonal anti-rat antibodies. The working concentration of the anti-HMGB1 antibody was 1:50.

### IP and Protein Acetylation Determination

The HMGB1-antibody was incubated with a tissue/cell extract to form an antibody/antigen complex in solution. The antibody/antigen complex was then pulled out of the sample using protein A/G-coupled agarose beads. The HMGB1 protein were separated by sodium dodecyl sulfate polyacrylamide gel electropheresis (SDS-PAGE) for western blot analysis using lysine-acetylation antibody and determine the ac-HMGB1.

### Isolation of Nucleus and Cytoplasm of RTECs

Tissue protein extraction reagent (T-PER) was used to isolate nuclear and cytoplasmic proteins. According to the manufacturer’s recommendations, protein yield from tissue or cell was harvested, washed twice and collected in ice-cold PBS and counted. The samples were pelleted by centrifugation at 2,000 ×*g* for 5 min and lysed in 1 mL T-PER for 5 min to obtain cytoplasmic proteins. The cell lysates were clarified by centrifugation at 14,000 ×*g* for 10 min and the supernatant was collected to obtain nuclear proteins, and the protein concentration (μg/million cells) was determined using the Pierce BCA Protein Assay. The nucleoprotein was detected by Histone H2A antibody. The cytoplasmic protein was detected by GAPDH antibody.

### Cell Culture and H/R Treatment of HK-2

The HK-2 cell line was purchased from Kunming Cell Bank (No. KCB200815YJ). This cell line is a clone of human renal cortical proximal tubular epithelial cells that are immortalized by HPV-16 E6/E7 and has various features of normal renal cortical proximal convoluted tubule epithelial cells. The cells were cultured in DMEM supplemented with 10% (v/v) heat-inactivated FBS and 1.0 mmol/L sodium pyruvate at 37°C in a humidified atmosphere containing 5% CO_2_. Based on our previous study, 6 h hypoxia (5% CO_2_, 1% O_2_, and 94% N_2_) followed by 2 h of re-oxygenation (5% CO_2_, 21% O_2_, and 74% N_2_) condition was chosen for H/R model reproduction.

### RNA Interference of SIRT1

**FIGURE 1:**
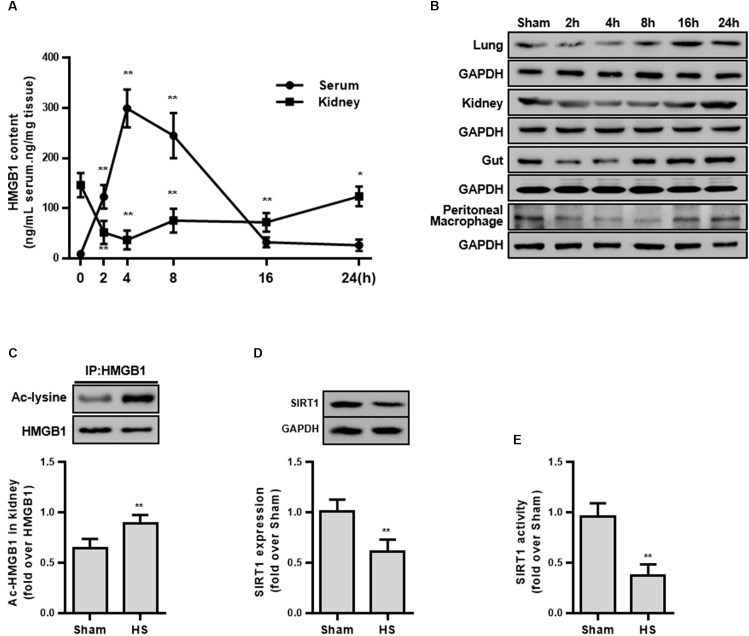
HMGB1 expression and acetylation, and SIRT1 protein and activity in HS rats. **(A)** Content of HMGB1 in serum and kidney tissue of rats following HS. *n* = 8. ^∗^ denotes *p* < 0.05, ^∗∗^ denotes *p* < 0.01 vs. 2 h following HS. **(B)** Expression level of HMGB1 in lung, kidney, small intestine, and peritoneal macrophages in rats following HS. **(C)** Ac-HMGB1 protein, **(D)** SIRT1 protein, and **(E)** SIRT1 activity in RTECs following HS. The ac-HMGB1 was determined by Western blot in purified HMGB1 after immunoprecipitation. Experimental values are compared with the sham group values. ^∗∗^ denotes *p* < 0.01 (*n* = 4). Ac, acetyl; HS, hemorrhagic shock.

The experiment was performed using HK-2 cells. HK-2 cells (1 × 10^6^) were grown in antibiotic-free DMEM in culture dishes for 24 h and were transfected with SIRT1-targeting siRNA or control siRNA using Opti-MEM I reduced serum media and Lipofectamine2000 according to the manufacturer’s instructions and the downregulated effect of SIRT1-targeting siRNA was confirmed in supplemented Figure 1. Twenty-four hours after transfection, the cells were exposed to normal cell culture medium. The cells were then collected and processed for immunoblotting to determine the level of SIRT1 and SIRT1 activity.

### Immunofluorescence Assay for the Intracellular Redistribution of HMGB1

The RTECs from rats or HK-2 cells were fixed with ice-cold 4% paraformaldehyde solution for 20 min and permeabilized in 0.2% Triton X-100 for 20 min after staining. Cells were then blocked with 1% bovine serum albumin for 30 min at room temperature. The primary rabbit polyclonal anti-HMGB1 (1:50 dilution) were incubated with the cells for >16 h at 4°C, and the secondary FITC-conjugated polyclonal goat anti-rabbit IgY s were added to the cells for 1 h at 37°C. Finally, 4′,6-diamidino-2-phenylindole (DAPI) was added for staining the nucleus. The cells were then observed and analyzed using a LSM 780 NLO laser scanning microscope confocal microscope system (Carl Zeiss, Jena, Germany).

### Determination of Acetylated Sites of HMGB1

Acetylated antibodies prepared for the five lysine residues sites (K28, K29, K30, K90, and K177) of HMGB1 were provided by ABclonal Biotechnology Co., Ltd. (Wuhan, Hubei, China). Briefly, after the HMGB1 protein was IP by commercial IP kit, the protein from the HK2 cells was incubated with acetyl-HMGB1 (ac-HMGB1) antibodies, was processed in Western Blot, and the acetylation level of different residue sites were then quantified.

### Measurement of Inflammatory Cytokines

The frozen supernatants samples of HK2 cells were analyzed to determine the concentrations of TNF-α, IL-1β, and IL-6 using commercially available ELISA kits according to the manufacturer’s recommendations.

### Analysis of Renal Function

Quantitative determination of BUN and Cr of the rats was done on Automatic Biochemical Analyzer (Olympus AU5400, Tokyo, Japan).

### Measurement of Apoptosis

Apoptosis of RTECs was measured using annexin V and propidium iodide kit. Apoptosis was reflected by annexin V positive ratio (%), which was determined using flow cytometry (BD FACSVerse, San Jose, CA) with methods described earlier (Wang et al., 2013) and a same gating strategy.

TUNEL staining of rat kidney tissue was carried out using a Promega apoptosis detection kit. Immunofluorescence for TUNEL staining was performed using FITC and 4′,6-diamidino-2-phenylindole (DAPI) and the glass was mounted with cover slips and imaged under a confocal microscope (LSM 780; Carl Zeiss, Oberkochen, Germany). TUNEL-positive apoptotic cells were counted in 10 random high-power fields (HPF; 300 cells each). Data are expressed as the number of apoptotic cells/HPF (magnification: 400×).

### Assay of SIRT1 Activity

Sirtuin 1 activity was determined in fresh kidney tissues or HK-2 cells using an anti-SIRT1 antibody. Activity of SIRT1 deacetylase was detected using SIRT1 Deacetylase Fluorometric Assay kits (Cyclex) as described previously (Zeng et al., 2016b). Briefly, renal tissue samples (50 mg) or RTECs were homogenized in 500 μL immunoprecipitation buffer. After immunoprecipitation of SIRT1, final reaction mixtures (50 μL) contained 50 mM Tris–HCl (pH 8.8), 4 mM MgCl2, 0.5 mM dithiothreitol, 0.25 mA/mL lysyl endopeptidase, 1 μM trichostatin A, 200 μM NAD+, and 5 μL extraction buffer. Fluorescence intensity at 350 nm/450 nm was measured using an Automatic Microplate Reader (Molecular Devices, Sunnyvale, CA, United States). The activity is presented as a relative value compared to the control group (sham).

### Transfection of Wild and Mutated SIRT1

HK2 cells were transfected with SIRT1, SIRT1^H363Y^, or control plasmids to mimic the overexpression of SIRT1, the inactivated form of SIRT1 using a transient transfection technical method. Twenty-four h after transfection, cells were washed and processed for immunoblotting and other studies described above. Two plasmids, Flag-SIRT1 and Flag-SIRT1^H363Y^ were designed by Addgene Corporation^1^^,^^2^ .

### Survival Study

After treatment and suturing, the survival time was recorded in rats (8 per group). All animals had *ad libitum* access to food and water. Apnea >1 min was considered to indicate death. Animals that survived longer than 48 h were euthanized by cervical dislocation.

### Statistical Analysis

The median survival time was analyzed using Kaplan-Meier plots and compared using the log-rank test. Other results are expressed as means ± standard deviation values, and statistical analysis was performed using one-way analysis of variance followed by Tukey multiple comparison test using SPSS software. Values were considered significant at *p* < 0.05.

## Results

### HMGB1 Is an Early Predictor of Hemorrhagic Shock

To explore whether HMGB1 is elevated during the early stage of HS, the serum HMGB1 content was detected and the result demonstrated a progressively increase following HS (Figure 1A). Unexpectedly, the HMGB1 content decreased in multiple organs (kidney, lung, and small intestine) (Figure 1B). Particularly in the kidney tissue, the HMGB1 content decreased by 94.6 ± 11.8 ng/mL at 2 h following HS (Figure 1A). This discrepancy of HMGB1 levels between serum and parenchyma organs prompted us to explore the potential mechanism. Since the largest decrease in HMGB1 was found in the kidney tissue, the kidney was selected for further study.

### Decreased SIRT1 Activity Accompanies Elevated HMGB1 Acetylation in Renal Tubular Epithelial Cells

As the secretion of HMGB1 is regulated by multiple post-transcriptional modifications including acetylation, we then determined the level of ac-HMGB1, which considerably increased in rat RTECs during HS (Figure 1C). As SIRT1 is an upstream regulator of HMGB1 and since we determined that reduced SIRT1 activity is involved in the pathogenesis in HS, we next determined the SIRT1 activity in RTECs. Consistent with our previous work (Zeng et al., 2016b), both SIRT1 expression and activity was considerably reduced in RTECs (Figure 1D,E). These data *in vivo* collectively suggest that reduced SIRT1 activity may lead to increased ac-HMGB1.

### Acetylation Modification Promotes the Nuclear-Cytoplasmic Translocation and Extracellular Secretion of HMGB1

To explore the mechanism of HMGB1 nuclear-cytoplasmic translocation, HMGB1 extracellular secretion, and the relationship between SIRT1 and HMGB1, we introduced an HK-2 hypoxia and re-oxygenation (H/R) model, which partially mimicked the condition of HS.

**FIGURE 2:**
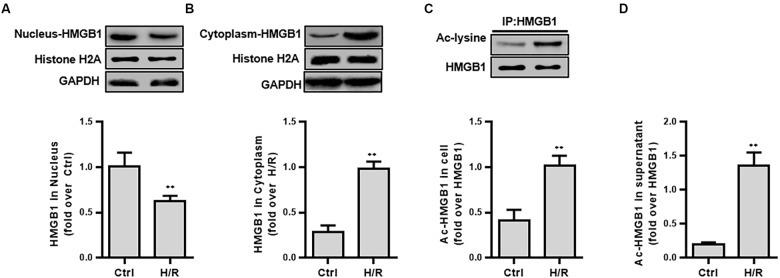
The expression and intracellular distribution of HMGB1 and ac-HMGB1 in HK-2 cell and supernatant following H/R. **(A)** Expression of HMGB1 in nucleus of HK-2 cells. **(B)** Expression of HMGB1 in cytoplasm of HK-2 cells. **(C)** Ac-HMGB1 in HK-2 cells. **(D)** Ac-HMGB1 in supernatant of HK-2 cells. ^∗∗^*p* < 0.01 compared with the value of control group, *n* = 4. HK-2, human kidney 2; Ac, acetyl; ctrl, control; H/R, hypoxia/reoxygenation.

First, we examined the protein expression and the acetylation level of HMGB1 in isolated components (nucleus, cytoplasmic protein) after H/R stimulation. H/R treatment lowered ac-HMGB1 levels in the nucleus to 38.70 ± 5.71% less that untreated HK-2 cell levels. In contrast, cytoplasmic ac-HMGB1 content increased by 8.46 ± 3.00 times that of the control group. Consistent with this result, we confirmed that ac-HMGB1 increased considerably in both whole cell lysate and cell culture supernatant, respectively (Figure 2).

**FIGURE 3:**
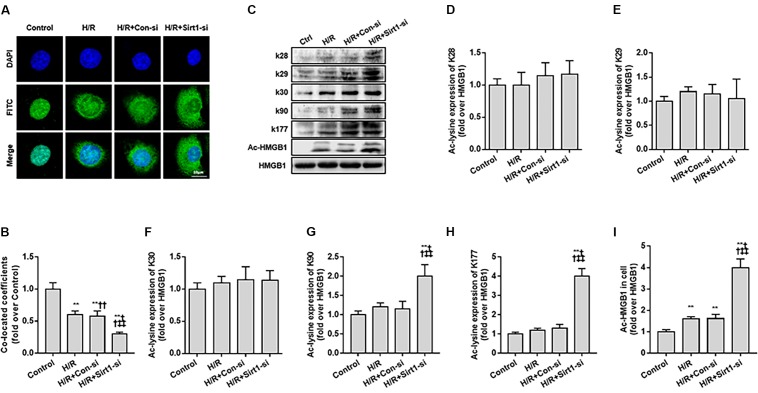
Translocation of acety-HMGB1 from the nucleus to cytoplasm in HK-2 cells following hypoxia and re-oxygenation. **(A)** Expression and intracellular distribution of HMGB1 in HK-2 cells. The translocation of HMGB1 was observed by FITC labeling under a confocal microscope (original magnification × 630). **(B)** Co-located coefficients of HMGB1 and nucleus in HK-2 cells. **(C)** Levels of HMGB1 acetylated at different lysine residues in HK-2 cells. **(D–I)** Relative gray values of HMGB1 acetylated at different lysine residues in HK-2 cells. Compared with control + H/R group, ^∗∗^ denotes *p* < 0.01; compared with H/R + Con-si group, ^††^ denotes *p* < 0.01; compared with H/R + Sirt1-si group, ^‡‡^ denotes *p* < 0.01; compared with SIRT1^H363Y^ group, ^&&^ denotes *p* < 0.01; *n* = 8. H/R, hypoxia/re-oxygenation.

Second, we used FITC-labeling to assess the nucleus to cytoplasm transport of ac-HMGB1 in HK-2 cell by confocal microscopy observation and employed a siRNA against SIRT1 (Supplementary Figure S1) for exact mechanism study. HMGB1, located in the nucleus under normal conditions (control group), is partly transferred to cytoplasm following H/R. The nucleus-to-cytoplasmic transport was accelerated with addition of SIRT1 siRNA (Figure 3A,B). As previous work identifies residues K28, K29, K30, K90, and K177 as potential acetylated sites of HMGB1 (Yang et al., 2004; Rabadi et al., 2015), we explored the exact HMGB1 acetylation sites during H/R using acetylated residue-specific HMGB1 antibodies. We found that the main HMGB1 acetylation lysine residues are K90 and K177, not K28, K29, K30 (Figure 3C–I).

**FIGURE 4:**
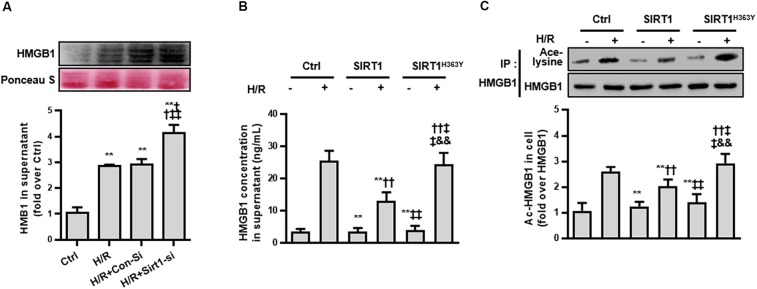
Release and acetylation of HMGB1 following hypoxia and re-oxygenation. **(A)** HMGB1 content in HK-2 cell culture supernatant following hypoxia and re-oxygenation. Compared with control group, ^∗∗^ denotes *p* < 0.01; compared with the H/R group, ^††^ denotes *p* < 0.01; compared with H/R + Con-Si group, ^‡‡^ denotes *p* < 0.01. *n* = 8. **(B)** HMGB1 content in HK-2 cell supernatant and **(C)** the acetylation level of HMGB1 in HK-2 cells. Compared with control + H/R group, ^∗∗^ denotes *p* < 0.01; compared with SIRT1 group, ^††^ denotes *p* < 0.01; compared with H/R + SIRT1 group, ^‡‡^ denotes *p* < 0.01; compared with SIRT1^H363Y^ group, ^&&^ denotes *p* < 0.01; *n* = 5.

Third, we measured the ac-HMGB1 in supernatant to evaluate the extracellular secretion level of HMGB1. We found that HMGB1, which maintains a very low level in normal conditions, was elevated over 2 times control levels after H/R treatment and was increased to 4 times control group levels when the SIRT1 gene was down-regulated by SIRT1 siRNA (*p* < 0.01; Figure 4A).

To ensure that the release of HMGB1 relies on a reduced SIRT1 deacetylase effect, we constructed a catalytic mutant of SIRT1 (SIRT1^H363Y^) lacking deacetylase activity. After overexpressing both SIRT1 and SIRT1^H363Y^ using a transient transfection technical method, the overexpression of SIRT1^H363Y^ (the deacetylase activity was deleted) attenuated the H/R-induced decrease in SIRT1 protein expression but not SIRT1 activity, which also lead to high acetylation and extracellular HMGB1 release (Figure 4B,C). These data indicate that SIRT1-mediated inhibition of HMGB1 extracellular secretion is dependent on its deacetylase activity, which may a new target for shock treatment.

### Deacetylation of HMGB1 Is a Therapeutic Target in the Early Stage of HS

**FIGURE 5:**
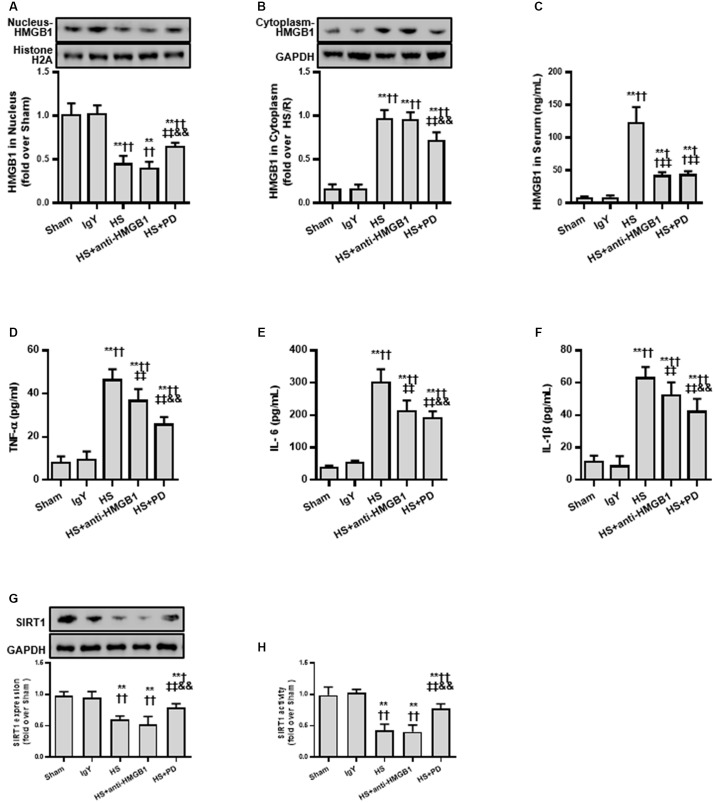
Effects of HMGB1 inhibition and SIRT1 activation on HMGB1 and inflammatory cytokine levels in RTECs following HS. **(A)** Nucleus HMGB1 expression in RTEC nucleus following HS. **(B)** Cytoplasm HMGB1 expression in RTEC cytoplasm following HS. **(C)** HMGB1 serum levels following HS. **(D–F)** Pro-inflammatory cytokines (TNF-α, IL-6, and IL-1β) following HS. **(G)** SIRT1 protein expression in renal cells following HS. **(H)** SIRT1 activity in renal cells following HS. Compared with sham group, ^∗∗^ denotes *p* < 0.01; compared with IgY group, ^†^ denotes *p* < 0.05, ^††^ denotes *p* < 0.01; compared with HS group, ^‡‡^ denotes *p* < 0.01; compared with HS + anti-HMGB1 group, ^&&^ denotes *p* < 0.01. *n* = 8. HS, hemorrhagic shock; PD, polydatin; IgY, immunoglobulin Y.

Based on the cell model study above, we next confirmed whether the SIRT1-mediated deacetylation of HMGB1 lies in the pathogenesis of HS using a typical HS rat model (Hwang et al., 2015; Rabadi et al., 2015). To test our hypothesis, we employed an HMGB1 neutralizing antibody and the putative inhibitor PD (as it activates SIRT1). We found that HMGB1 neutralizing antibody decreased HS-mediated elevation of serum HMGB1 (Figure 5C) and decreased other inflammatory factors (Figure 5D–F). However, the neutralizing antibody could not reduce the nucleus to cytoplasm shuttle of HMGB1 nor its acetylation level (Figure 5A,B). Interestingly, PD enhanced both protein expression (Figure 5G) and activity of SIRT1 (Figure 5H). Moreover, PD not only decreased HMGB1 expression, but also attenuated its nucleus to cytoplasm shuttle and, importantly, reduced HMGB1 acetylation (Figure 5).

**FIGURE 6:**
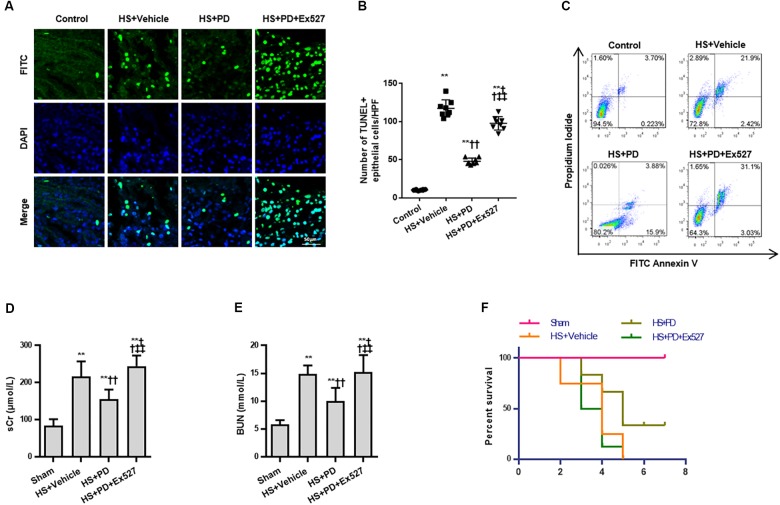
Effects of SIRT1 activation on apoptosis, renal function and survival time of rats following HS. **(A)** HS-mediated RTEC apoptosis. The TUNEL positive cells were recognized as apoptotic cells in kidney tissue. **(B)** Statistical analysis of the TUNEL results. **(C)** Apoptosis determination of rat RTECs following HS. Apoptosis was reflected by annexin V positive ratio (%). **(D,E)** Renal function was reflected by serum Cr and BUN following HS. **(F)** Survival curve analysis of rats following HS. The median survival time was analyzed using Kaplan-Meier plots and compared using the log-rank test. Compared with sham group, ^∗∗^ denotes *p* < 0.01; compared with HS + Vehicle group, ^††^ denotes *p* < 0.01; compared with HS + PD group, ^‡‡^ denotes *p* < 0.01; *n* = 8. HS, hemorrhagic shock; PD, polydatin; sCr, serum Cr; FITC, fluoresceine isothiocyanate; TUNEL, terminal deoxynucleotidyl transferase-mediated dUTP-biotin nick end labeling.

Due to the suppressive effects of SIRT1 on HMGB1, we finally assessed the effect of SIRT1 activation on RTEC apoptosis, renal function, and survival time following HS. Both TUNEL (Figure 6A,B) and flow cytometry (Figure 6C) results showed that RTEC apoptosis was increased with increased TUNEL positive cells and annexin V positive ratio following HS. Moreover, renal function were deteriorated by increased serum creatinine (sCr) and BUN. Importantly, SIRT1 activation with PD strongly reduced RTEC apoptosis with decreased TUNEL positive cells and annexin V positive ratio, improved renal function with reduced sCr and BUN (Figure 6D,E), and extended the animals’ survival time from HS (Figure 6F).

## Discussion

**FIGURE 7:**
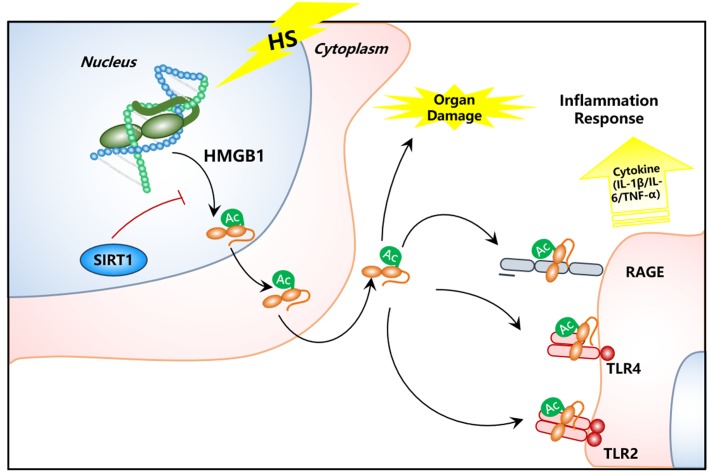
Schematic image of SIRT1 activation for deacetylation of HMGB1 and suppression of inflammatory signaling in kidney cells for the treatment of HS.

In this study, we found increased HMGB1 content in serum of HS rats. In contrast, the HMGB1 expression in some parenchymal organ cells (lung, kidney, and gut) unexpectedly decreased after HS, especially in kidney tissue. Decreased HMGB1 levels in RTECs was accompanied with elevated nucleocy-to-plasmic HMGB1 translocation, increased supernatant concentration of HMGB1 protein, and increased HMGB1 acetylation as shown in our *in vivo* HS rat model and *in vitro* H/R HK-2 cell model, respectively. However, when SIRT1 activity was enhanced, both the nucleus to cytoplasm trafficking and acetylation of HMGB1 was reduced. This study provides direct evidence for a HMGB1 release mechanism by kidney cells in the early stages of HS. To the best of our knowledge, this is the first study that demonstrates the exact mechanism of both intracellular distribution and extracellular secretion of HMGB1 from parenchymal organ in HS (Figure 7). Thus, targeting deacetylated HMGB1 and subsequently inhibiting its nucleo-cytoplasmic translocation may present a promising treatment for HMGB1-mediated inflammation in HS.

High mobility group box 1 was first reported as a late mediator of lethality in sepsis (Wang et al., 1999; Yang et al., 2004). Plasma HMGB1 is significantly increased during the early stages of trauma, burns, and HS patients (Ombrellino et al., 1999; Cohen et al., 2009; Peltz et al., 2009; Lantos et al., 2010). HMGB1 is recognized as an alarmin, capable of mediating damage signals and stimulating an immune response. In both HS and sepsis models, elevated HMGB1 enhanced the inflammatory response (Ombrellino et al., 1999; Cohen et al., 2009; Peltz et al., 2009; Lantos et al., 2010). Due to the elevated HMGB1 content and its harmful effect in both IR-induced renal cell injury (Li et al., 2011) and HS-induced gut barrier dysfunction (Yang et al., 2006), anti-HMGB1 neutralizing antibody have shown an effective therapeutic roles. However, in this study, we confirmed that the HMGB1 content in the serum is highly elevated in early stages of HS. To further explore the mechanism underlying enhanced HMGB1 expression, we next investigated the source and stimulus of plasma HMGB1 during the early stages of HS.

In general, HMGB1 is released from an intracellular location to the extracellular space via two routes: active secretion following cell activation, and passive release following cell necrosis, but not apoptosis. HMGB1 may be secreted from macrophages, small intestine, and gut tissue in sepsis, ischemia, and traumatic injury/HS, respectively (Tsung et al., 2007; Vande et al., 2011; Sodhi et al., 2015). Interestingly, general levels of HMGB1 were decreased from not only in immune cells (macrophages in the peritoneum), but also in various organ cells (lung, kidney, and small intestine) in HS rat models. In the present study, we chose to focus on exploring HMGB1 secretion from RTECs because HMGB1 protein in the kidney tissue was most obviously decreased during HS.

High mobility group box 1 hyperacetylation has been found to be a seminal event prior to its secretion in some disease models. In a hepatic ischemia/reperfusion injury model, enhanced acetylation promoted HMGB1 translocation and release in liver cells (Tsung et al., 2007; Vande et al., 2011; Sodhi et al., 2015). Thus, the deacetylate modification of HMGB1 represents a potential method of inhibiting HMGB1-mediated inflammation. Of note, the deacetylation effect of HMGB1 and its possible mechanism in HS have not been demonstrated before, especially in kidney cells. We have previously shown that deacetylase SIRT1 protein level and deacetylation activity were significantly decreased in various organs (kidney and small intestine) during severe HS (Zeng et al., 2015, 2016a). However, the mechanism of SIRT1 downregulation in HS remains to be explored. In this study, we found that 4 h after H/R, SIRT1 activity decreased, and ac-HMGB1 levels increased in rat RTECs. We observed ac-HMGB1 translocation from the nucleus to cytoplasm and secretion to extracellular space in isolated HK-2 under H/R using Western blot, FITC-labeling, and ELISA technique, respectively. SIRT1-HMGB1 immunoprecipitation, SIRT1-targeting siRNA, and SIRT1 mutations were used to confirm SIRT1’s-regulation of HMGB1 release from RTECs in HS. Activation of SIRT1 blunted HMGB1 release, salvaged renal function, and lengthened animal survival from HS. However, SIRT1 overexpression has no significant inhibitory effect on HMGB1 under physiological conditions, probably because SIRT1 regulates HMGB1 depending on the specific disease environment. Our findings demonstrate that the attenuation of SIRT1-induced deacetylation of HMGB1 is an upstream signaling pathway of the HMGB1-associated inflammatory response in the pathogenesis of HS. Our findings suggest that blocking the acetylation of HMGB1 during the initial stage of HS rather that neutralize HMGB1 is a promising method for future HS-induced AKI treatment. Since the response downstream of deacetylase SIRT1 is versatile (deacetylate SOD2 for oxidative stress attenuation, deacetylate p53 for apoptosis inhibition, and deacetylate HMGB1 for inhibition of the inflammatory response, etc.), it is not difficult to speculate that SIRT1 has great potential in HS treatment. Furthermore, some selective/physiological SIRT1 agonists have been successively discovered, such as resveratrol (Wang et al., 2015), polydation (Zeng et al., 2015), and melatonin (Wu et al., 2008). These findings provide strong evidence for the role of SIRT1 activators in the application of clinical HS and other diseases.

The direct evidence of SIRT1-deacetylated HMGB1 was first identified in ischemic conditions (Rabadi et al., 2015). When IP ac-HMGB1 was incubated with SIRT1, HMGB1 acetylation was considerably decreased. Moreover, four lysine residues (55, 88, 90, and 177) of HMGB1 were confirmed by proteomic analysis. SIRT1 activation led to decreased HMGB1 acetylation, and reduced tubular damage (Rabadi et al., 2015). In addition, SIRT1 has been reported to form a stable complex with HMGB1 and to directly interact with HMGB1 via its N-terminal lysine residues (28–30) in murine macrophage RAW264.7 cells, thereby inhibiting HMGB1 release to improve survival in an experimental model of sepsis (Hwang et al., 2015). In this study, we found that during the early stage of HS, both the SIRT1 expression and activity were reduced in RTECs. To explore the underling mechanism of HMGB1 in HS, we focused on the kidney, which is rich in SIRT1 content. As expected, the decreased SIRT1 activity concomitantly increased the acetylation of HMGB1 and nucleus-to-cytoplasmic translocation and extracellular secretion into the milieu of RTECs. Additionally, SIRT1 has been found to physically interact with HMGB1 protein in HS or hypoxia-reoxygenation conditions. Utilizing the genetic downregulation or pharmacological inhibition of SIRT1 described earlier, we further confirmed the deacetylation of SIRT1 on HMGB1. These results suggest that SIRT1-mediated deacetylation of HMGB1 would be a promising therapeutic strategy for HS treatment, especially during the initial stages, since the excessive inflammation is the main cause of post-shock complications. Previous reports show that the main deacetylation site of HMGB1 is lysine residues (28–30) (Hwang et al., 2015) in sepsis-induced murine macrophage or lysine residues (55, 88, 90, and 177) (Rabadi et al., 2015) in ischemic endothelial cells. Our recent published paper showed that the main acetylated sites of HMGB1 in sepsis induced AKI is K28–30 (Wei et al., 2018). In this study, we used a series of commercial kits targeting the individual sites (28–30, 90, and 177, respectively). Similarity, we confirmed that the attenuation of SIRT1-induced deacetylation of HMGB1 is also lying in the pathogenesis of HS induced AKI. In contrast to our previous work, we found that the main lysine residues of HMGB1 are K90 and K177, not K28-30 in H/R HK-2. Furthermore, our study provide a detailed mechanism that SIRT1 inactivation promote the acetylation of HMGB1, leading to its nucleus-to-cytoplasm translocation, and finally the presumed extracellular secretion with increased serum concentration. Our data collectively indicates that the deacetylation modification of HMGB1 may differ between different cell types or stress conditions, which poses a challenge for the precise regulation of HMGB1.

There are several limitations in our study. First of all, we only detected the increased serum HMGB1 content following HS, the evidence of HMGB1 secretion from renal epithelial cell is yet to be provided. Secondly, more vital organs such as liver and small intestine might also be the main resource of ac-HMGB1 in the pathogenesis in HS and that needs to be confirmed. Thirdly, the role of the attenuation of SIRT1-induced HMGB1 deacetylation may also be differed in various stage in HS, especially in the later period with immunosuppressive state, which is expected to test in our and others’ later work.

Collectively, our results demonstrate that serum HMGB1 increases in the early stages of HS, which might come from immune cells and various visceral organ cells as well. We provide evidences for HMGB1 release from kidney cells and this release facilitates the genesis of kidney injury in HS. Considering this concept, we postulate that secretion of HMGB1 not only leads to an increase of serum HMGB1 level, but also evokes inflammatory cytokine storm in local organs and facilitates the genesis of organ failure. These findings open a new approach for the treatment of shock and multiple organ failure in future medical research.

## Conclusion

In present study, we found that: (1) elevated HMGB1 protein is a critical process of HS-induced inflammation; (2) the increased HMGB1 content is at least partially due to the secretion of RTECs; (3) acetylation modification of HMGB1 is the prior seminal event for the nucleus-to- cytoplasmic translocation of HMGB1 and subsequent secretion; (4) The acetylated/deacetylated sites of HMGB1 varied due to different microenvironment; and(5) SIRT1-mediated deacetylation of HMGB1 may represent a promising therapeutic target for shock treatment.

## Ethics Statement

The present study was carried out in strict accordance with the recommendations in the Guide for the Care and Use of Laboratory Animals (US National Institutes of Health, Bethesda, MD, United States). The study protocol was approved by the Committee on Ethics in Animal Experiments of the Southern Medical University.

## Author Contributions

SX, ZZ, and KZ conducted the *in vivo* and *in vitro* experiments, conceived of and designed the study, and drafted the manuscript. MZ and QH participated in interpretation of the studies and discussed the results of experiments. XD collected and analyzed the clinical data. YG, JL, and WH prepared the figures and tables. All authors approved the final manuscript.

## Conflict of Interest Statement

The authors declare that the research was conducted in the absence of any commercial or financial relationships that could be construed as a potential conflict of interest.

## Footnotes

1 http://www.addgene.org/1791/

2 http://www.addgene.org/1792/

## Supplementary Material

FIGURE S1 The expression of SIRT1 in HK-2 cells after siRNA transfection. Compared with the Control group, ^∗∗^ denotes *p* < 0.01; compared with the H/R group, ^††^ denotes *p* < 0.01; compared with the H/R + Con-Si group, ^‡‡^ denotes *p* < 0.01; *n* = 8. H/R, hypoxia/re-oxygenation; si, siRNA; con, control; GAPDH, glyceraldehyde 3-phosphate dehydrogenase.

## Abbreviations

ac-HMGB1: acetylated HMGB1
ac-lysine: acetylated lysine
AKI: acute kidney injury
APACHE II: acute physiology and chronic health evaluation II
BCA: bicinchoninic acid
BUN: blood urea nitrogen
Cr: creatinine
DMEM: Dulbecco’s modified Eagle’s medium
ELISA: enzyme-linked immunosorbent assay
FBS: fetal bovine serum
FITC: fluorescein isothiocyanate
GAPDH: glyceraldehyde 3-phosphate dehydrogenase
H/R: hypoxia-reoxygenation
HepG2: liver hepatocellular carcinoma
HK-2: human renal proximal tubular epithelial cell line
HMGB1: high mobility group box 1
HS: hemorrhagic shock
i.v.: intravenous
IgY: immunoglobulin Y
IL-1β: interleukin-1β
IL-6: interleukin-6
IP: immunoprecipitated
IP: immunoprecipitation
IR: ischemia reperfusion
MAC: minimum alveolar concentration
MAP: mean arterial pressure
NF-κB: nuclear factor kappa-light-chain-enhancer of activated B cells
PBS: phosphate buffer saline
PD: polydatin
PVDF: polyvinylidene fluoride
RIPA: radio-immunoprecipitation assay
RTEC: renal tubular epithelial cell
SBP: systolic blood pressure
SDS-PAGE: sodium dodecyl sulfate polyacrylamide gel electrophoresis
siRNA: small interference RNA
SIRT1: sirtuin 1
SOD2: manganese superoxide dismutase 2
TBST: Tris-buffered saline
TNF-α: tumor necrosis factor-α
TUNEL: terminal deoxynucleotidyl transferase dUTP nick end labeling
