# Supplementary figures and images for: Evidence for SIRT1 Mediated HMGB1 Release From Kidney Cells in the Early Stages of Hemorrhagic Shock
Source: Front Physiol. 2019 Jul 5;10:854. doi: 10.3389/fphys.2019.00854 (PMC6625367; doi:10.3389/fphys.2019.00854)

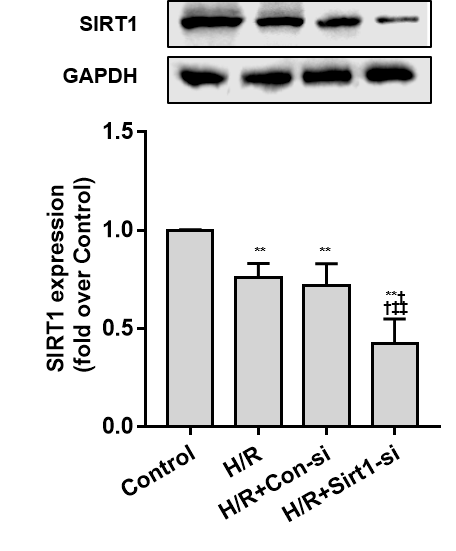

Supplement: FIGURE S1 — The expression of SIRT1 in HK-2 cells after siRNA transfection. Compared with the Control group, ∗∗ denotes p < 0.01; compared with the H/R group, †† denotes p < 0.01; compared with the H/R + Con-Si group, ‡‡ denotes p < 0.01; n = 8. H/R, hypoxia/re-oxygenation; si, siRNA; con, control; GAPDH, glyceraldehyde 3-phosphate dehydrogenase. [file Image_1.TIF]
